# Retinal prostheses: progress toward the next generation implants

**DOI:** 10.3389/fnins.2015.00290

**Published:** 2015-08-20

**Authors:** Diego Ghezzi

**Affiliations:** Medtronic Chair in Neuroengineering, Center for Neuroprosthetics, Interfaculty Institute of Bioengineering, School of Engineering, École Polytechnique Fédérale de LausanneLausanne, Switzerland

**Keywords:** vision, retinal prosthesis, photovoltaic stimulation, thermal stimulation, ultrasonic stimulation

## Abstract

In the last decade, various clinical trials proved the capability of visual prostheses, in particular retinal implants, to restore a useful form of vision. These encouraging results promoted the emerging of several strategies for neuronal stimulation aiming at the restoration of sight. Besides the traditional approach based on electrical stimulation through metal electrodes in the different areas of the visual path (e.g., the visual cortex, the lateral geniculate nucleus, the optic nerve, and the retina), novel concepts for neuronal stimulation have been mostly exploited as building blocks of the next generation of retinal implants. This review is focused on critically discussing recent major advancements in the field of retinal stimulation with particular attention to the findings in the application of novel concepts and materials. Last, the major challenges in the field and their clinical implications will be outlined.

## Introduction

Low vision and blindness can result when any step of the visual pathway (the cornea, the lens, the retina, the optic nerve, the thalamus, and the visual cortex) is altered or sustains damage. In the recent years, a great attention has been dedicated to inherited retinal dystrophies, one of the major causes of adult blindness in industrialized countries, causing the malformation or progressive degeneration, and death of retinal photoreceptors (Wright et al., 2010). Retinal dystrophies, such as *Retinitis pigmentosa* (Hartong et al., 2006), and macular degeneration (Swaroop et al., 2009), are a complex trait that is influenced by many genes together with many environmental factors; indeed, it is considered the most genetically heterogeneous disorder in humans. Several approaches have been described for the restoration of sight due to photoreceptor degeneration (Jacobson and Cideciyan, 2010; Busskamp et al., 2012), some of them have also reached human clinical trials. They include attempts to slow down the degeneration process by pharmacotherapy (Frasson et al., 1999), to correct the defect by gene supplementation therapy (Smith et al., 2012), to replace lost photoreceptors by means of transplantation of retinal sheets (Seiler and Aramant, 2012) or photoreceptor precursors (MacLaren et al., 2006; Pearson et al., 2012), to sensitize to light the remaining retinal cells by means of optogenetics (Busskamp et al., 2010; Busskamp and Roska, 2011), or to bypass photoreceptors with visual prostheses (Zrenner, 2013).

After the development and successful clinical application of cochlear implants (Rauschecker and Shannon, 2002; Clark, 2006), neural prostheses were exploited in the treatment of diverse acquired or hereditary neural defects including visual impairments. However, restoring vision with bionic devices can be much more complicated. The idea of probing electrical stimulation to restore vision was first reported by Foerster (1929) and subsequently explored by coupling surface electrodes to the visual cortex (Brindley and Lewin, 1968; Dobelle and Mladejovsky, 1974; Dobelle et al., 1974). Some of these approaches were even tested in human volunteers reporting perception of phosphenes. Morevoer, early attempt to retinal stimulation (Dawson and Radtke, 1977) were also exploited following the demonstration that trans-ocular retinal stimulation can induce the perception of phosphenes in both normal subjects (Carpenter, 1972) and blind people affected by *Retinitis pigmentosa* (Potts and Inoue, 1969). Since loss of vision can results from the alteration of any element of the visual pathway from the eye to the visual cortex in the brain, the approaches currently exploited as visual prostheses have been classified in cortical (Schmidt et al., 1996; Maynard et al., 1997; Normann et al., 1999; Rousche and Normann, 1999), optic nerve (Veraart et al., 2003; Fang et al., 2005; Sakaguchi et al., 2012), or retinal (da Cruz et al., 2013; Stingl et al., 2013) implants depending on their position in the visual path (Winter et al., 2007). Retinal prostheses are designed to treat diseases affecting photoreceptors, whereas optic nerve and cortical prostheses could theoretically address these conditions plus those occurring further along the visual path (e.g., glaucoma, diabetic retinopathy, severe optic atrophy, and traumatic damages) (Merabet et al., 2005). Retinal implants quickly became the preferred strategies, because they can benefit from the natural information processing along the visual path. On the other hand, cortical prostheses are hampered by the difficult to communicate directly at the highest level of the visual information processing.

Retinal implants can be classified on the basis of two distinctive criteria, their position with respect to the retina or their functional principle. Two kind of retinal implants have been proposed on the basis of the first criterion: epi-retinal and sub-retinal. In the epi-retinal configuration, the prosthesis interfaces directly the ganglion cell layer, whereas in the sub-retinal configuration the implant is positioned behind the retina in place of photoreceptors. Based on the second criterion, retinal prostheses can be identified as micro-photodiode arrays (MPDAs) or micro-electrode arrays (MEAs). MEA-based devices consist of an implanted electrode array physically connected to a multichannel stimulator. Images are collected by a digital camera and processed by an image-processing unit, which in turn develops and generates a specific stimulation pattern wirelessly transmitted to the implanted chip. Recently, these retinal implants have been developed mainly by three research consortium: Boston Retinal Implant Project (Boston, MA, USA), Second Sight® Medical Products Inc. (Symlar, USA), and Pixium Visium SA (Paris, France). The first targets the subretinal implant location, while the latter two target an epiretinal implant location. On the contrary, MPDAs have been exploited mainly by two consortiums: Optobionics (Chicago, USA) and Retina Implant AG (Reutlingen, Germany). In this configuration, the light impinging the retina is converted by the photodiodes in electrical stimuli delivered to the inner retina through metal electrodes. In general, they are designed to be positioned in the sub-retinal space, thus providing functional replacement of photoreceptors, representing the earliest possible level, thus reducing the level of signal processing needed. On the same framework, the unit of Grabiel A. Silva is developing a new photo-detector technology approaching the natural photoreceptors with nanoscale control of topography (Khraiche et al., 2011). This technology employs semiconductor vertical nanowires that promise to be high sensitivity, low power, and broadband photo-detectors.

## Novel interfaces and stimulation paradigms

### Photovoltaic stimulation

Current techniques employed in the fabrication of retinal prostheses must provide control signals and/or power to the implanted chip. This is typically solved via transdermal inductive coils that deliver energy and signals via intraocular cables to the implanted array. In general, this limits the number of electrode that can be addressed, entails the need of sealed case for the implanted electronic circuit, and requires the implementation of complex surgical procedures (Zrenner, 2012).

Photovoltaic retinal stimulation was envisaged in the middle of the 50's when a photosensitive selenium cell placed behind the retina of a blind patient resulted in the detection of phosphenes when illuminated (Tassicker, 1956). This concept was explored again in the early 90's by Alan Chow and associated that proposed the implantation of a photovoltaic semiconductor-based MPDA with capacitive electrodes (Chow et al., 2004, 2010), the artificial silicon retina (ASR, Optobionics). After preliminary studies in rabbits (Chow and Chow, 1997) and cats (Chow et al., 2001), in June 2000 and July 2001, six *Retinitis pigmentosa* patients were implanted with the ASR chip in a pilot feasibility and safety study with a follow up of 6 months (3 patients) and 18 months (3 patients) (Chow et al., 2004). As result of the trial, all patients observed vision improvements of the implanted eye in retinal areas both adjacent to and distant to the implant that included the macular area. These vision changes consisted of subjective and objective improvements of complex visual function such as visual acuity, and the perceptions of color, contrast, and darkness. Because of the huge improvements in visual functions and because of these improvements involved locations distant from the implant, they were unlikely due to an acute effect from ASR electrical stimulation. In addition, improvements also did not occur immediately, but 1 week to 2 months later. The findings suggested that a generalized neurotrophic effect might have resulted from ASR electrical stimulation.

**Figure 1:**
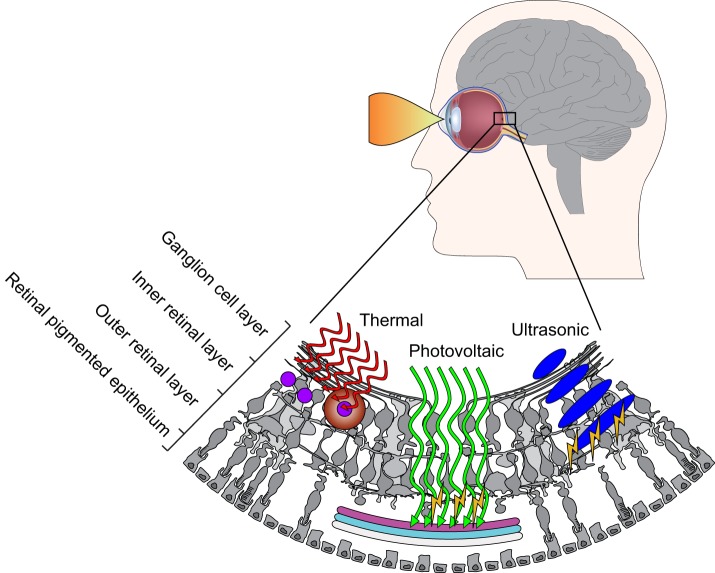
**Novel strategies for retinal stimulation**. Photothermal stimulation *left* requires photo-absorber (e.g., black μ-particles) that absorb light energy and transfer it to heat, which in turn activates the cells in their near vicinity. Photovoltaic stimulation *middle* requires photoresponsive surfaces (silicon, conjugated polymers, etc.) inducing capacitive stimulation upon light absorption and charge generation. In ultrasonic stimulation *right*, ultrasonic waves are transmitted into the eye and interfere to create a projected pattern for exciting retinal neurons.

In the recent years, the group of Daniel Palanker demonstrated the possibility to restore light responses with a photovoltaic sub-retinal implant (Mathieson et al., 2012; Mandel et al., 2013). Based on a similar concept of the ASR, this photovoltaic sub-retinal implant allows the restoration of light sensitivity in blind rats with photoreceptor degeneration. Moreover, they demonstrated the capability to restore visual acuity up to 0.47 cycles per degree, roughly corresponding to 20/250 when theoretically scaled to the dimension of the human eye. Moreover, this limit appears to be closely related with the pixel size; thus a further reduction of the pixel size could support an even higher visual acuity. In the same years, two independent groups pioneered the concept of photovoltaic stimulation with conjugated polymers (Ghezzi et al., 2013; Gautam et al., 2014). The advantage in using conjugated polymers in retinal implants consists in a large absorption coefficient allowing for the fabrication of thin, lightweight, and pliable structures (Figure 1). Moreover, the solution-processable films can be patterned on flexible and conformable substrates for a better match with the retina mechanics. These devices have been validated *in vitro* with retinal explants from light-induced degenerated rats and chicken embryos. Lately, Yael Hanein proposed the use of a semiconductor nanorod-carbon nanotube pair for photovoltaic stimulation of the retina providing a highly efficient photosensitivity (Bareket et al., 2014).

### Photo-thermal stimulation

Direct infrared neural stimulation (INS) has been recently introduced has alternative to optogenetic methods for light-tissue interaction (Wells et al., 2005). The principle of INS stimulation consists in the use of infrared laser pulses tuned to water absorption peaks; infrared light absorption by the tissue's water content is putatively responsible of causing rapid membrane capacitance changes leading to photo-thermally induced neural excitation (Shapiro et al., 2012). To adapt this concept to vision restoration (Figure 1), water absorption was replaced by photo-absorbers (e.g., black micro-particles) scattered in close proximity of the target cells (Farah et al., 2013). In addition, the photo-thermal approach was also demonstrated be able not only to induce neuronal excitation but also neuronal silencing upon prolonged illumination (Yoo et al., 2014). In the framework of vision restoration, photo-thermal modulation could now represent a novel strategy to induce both excitation and inhibition in the retinal circuitry. In addition, these effects are now taken in consideration also in the photovoltaic retinal prostheses, where light absorption (e.g., by conjugated polymers) may induce also photo-thermal responses (Martino et al., 2015).

In summary, photo-thermal modulation based on heating of exogenous photo-absorbers is a potential high-resolution optical stimulation approach; however, it is still in its early stage of biophysical and physiological characterization.

### Ultrasonic stimulation

Ultrasound waves were firstly introduced as a tool for retinal stimulation in 2012 by Shy Shoham, who envisaged a non-invasive retinal device sculpting the ultrasonic field to obtain an efficient neuro-stimulation (Naor et al., 2012). The concept behind the acoustic retinal prosthesis (Figure 1) consists in an external camera with an image-processing unit that transmit pattern information to a multi-element phases array located externally to the cornea with a coupling gel. Waves penetrate the eye and create a projected pattern for exciting retinal neurons. Recent studies from the group of William J. Tyler confirmed that focused ultrasonic pulses efficiently stimulate cortical neurons *in vitro* (Tyler et al., 2008), *in vivo* (Tufail et al., 2010), and in humans (Legon et al., 2014). Retinal stimulation with acoustic waves was demonstrated *in vivo* (Naor et al., 2012) and with retinal explants (Menz et al., 2013). In the latter report, authors demonstrated that ultrasound activated in part interneurons beyond photoreceptors; these evidences suggest ultrasonic stimulation as a valuable technology in case of photoreceptor degeneration.

Although promising, many questions about this technological framework still remain open. The mechanism of biological transduction of ultrasonic stimulation is largely unknown; therefore it is not yet clear if this effect is ubiquitous or delivered only to specific cell types. Moreover, additional investigations are required to understand the long-term tolerability of ultrasonic stimulation.

## Challenges in materials and fabrication

First, any prosthesis should be considered biocompatible, thus minimizing or avoiding problems related to tissue encapsulation and cellular/immune response. On the other hand, it should be mechanically stable once implanted, with a lifetime compatible with the human life (order of decades). In general, the pure biocompatibility of implants described so fare are good. However, in addition to biocompatibility issues related to the use of foreign materials in the host eye, toxic effects at the pixel level must be considered. In fact, cellular-electrode interface is an argument of intense research in the recent years, in terms of chemical composition, physical, and mechanical properties, long-term stability, and charge transfer from the electrode to the target tissue. Material selection plays a critical role. As an example, carbon-based materials (e.g., conjugated polymers, carbon nanotubes, graphene) have now been explored as the working element in a vast array of bionic devices, thus becoming a valuable solution for the next generation of implants (Fattahi et al., 2014).

A second challenge in retinal prosthetics consists in the design of prostheses allowing for the coverage of a wide area. Surface coverage directly affects the size of the field of view restored in the blind patient, thus it became of primary importance to guarantee an enlargement of the central visual field enough to allow reasonable mobility. However, because of technological and surgical limits, Argus II™, the largest prosthesis implanted in humans sizes approximately 3 × 5 mm thus covering 11° × 19° in the visual field (~22° diagonally). Other prostheses typically are in the range of 1-3 mm. Investigation on normal individuals under pixelated vision condition indicated that 30° of visual field could provide adequate mobility skills (Cha et al., 1992; Dagnelie et al., 2007). Preliminary attempts in providing wide-field retinal prostheses have been proposed (Ameri et al., 2009; Waschkowski et al., 2014); however, implantation of a large electrode array brings on new challenges that are still unmet. As the area of the implant increase, the conformity of the array to the eye curvature became important to minimize the relative distance between the electrodes and the retinal surface. This is an important parameter in order to minimize the strength of the electric pulse and increase the resolution in stimulation. In addition, the ability to fold prior to surgical insertion then unfold it inside the eye chamber is mandatory in order to insert the prosthesis through a scleral incision. The selection of the proper materials and fabrication strategies plays a major role toward this goal.

Complementary to the enlargement of the visual field, the design of retinal prosthesis with high pixel density matches the need of restoring a useful visual acuity in the blind patient. However, increasing coverage together with pixel density leads to the increase of the number of pixels, possibly introducing significant technological problems (Palanker et al., 2005). While micro-fabrication techniques allow for the realization of high dense MEAs, however electrode routing and the size of the connecting cable with the extra-ocular device still represent the major limitation. In this perspective, photovoltaic stimulation represents a powerful strategy to avoid intra-extra ocular connection and simplify the surgical procedure. However, once again, photovoltaic elements needs to be fabricated on flexible wide-field substrates; thus requiring the implementation of conformable materials and fabrication techniques.

## Conclusion

Retinal stimulation represents a real solution for restoring vision in blind people. The clinical trials with conventional retinal prostheses already demonstrated that tangible results can be obtained in everyday life. However, the goal of “restoring vision” is still unreached. The challenges behind making retinal stimulation a valuable solution for blindness must be faced by an interdisciplinary activity including experts in material science, micro-fabrication, neuroscientists, and clinicians.

### Conflict of interest statement

The author declares that the research was conducted in the absence of any commercial or financial relationships that could be construed as a potential conflict of interest.
